# Self-motion evokes precise spike timing in the primate vestibular system

**DOI:** 10.1038/ncomms13229

**Published:** 2016-10-27

**Authors:** Mohsen Jamali, Maurice J. Chacron, Kathleen E. Cullen

**Affiliations:** 1 Department of Physiology McGill University, Montreal, Quebec, Canada H3G1Y6

## Abstract

The accurate representation of self-motion requires the efficient processing of sensory input by the vestibular system. Conventional wisdom is that vestibular information is exclusively transmitted through changes in firing rate, yet under this assumption vestibular neurons display relatively poor detection and information transmission. Here, we carry out an analysis of the system's coding capabilities by recording neuronal responses to repeated presentations of naturalistic stimuli. We find that afferents with greater intrinsic variability reliably discriminate between different stimulus waveforms through differential patterns of precise (∼6 ms) spike timing, while those with minimal intrinsic variability do not. A simple mathematical model provides an explanation for this result. Postsynaptic central neurons also demonstrate precise spike timing, suggesting that higher brain areas also represent self-motion using temporally precise firing. These findings demonstrate that two distinct sensory channels represent vestibular information: one using rate coding and the other that takes advantage of precise spike timing.

## Abstract (web summary)

Early vestibular pathways are thought to code sensory inputs regarding self-motion via changes in firing rate. Here, the authors record from both regular and irregular afferents in macaques, and find both irregular afferents and central neurons also represent self-motion via temporally precise spike timing.

Understanding the set of transformations by which the brain processes incoming sensory input to ensure accurate perception and behaviour remains a central problem in neuroscience. The vestibular system provides information about our self-motion and spatial orientation relative to the world that is required for ensuring gaze stability, balance and postural control during everyday life. This essential sensory system comprises two classes of primary afferents that differ in their morphology and response dynamics (reviewed in ref. 1). Historically, these two afferent classes have been termed regular and irregular because the distribution of resting discharge variability is bimodal. The evolution of amniotes from amphibian-like animals was accompanied by the appearance of a new type of vestibular receptor cell that preferentially supplies irregular afferents (reviewed in ref. 2). It has been hypothesized that the relatively late appearance of the type I hair cell in evolution demonstrates a neural adaptation to changes in natural stimulus statistics following the transition from a water-based to a land-based environment (reviewed in refs 1, 3). However, this proposal is at odds with recent findings that despite displaying higher sensitivity, irregular afferents are actually worse at detecting and transmitting information about self-motion than their regular counterparts^4^,^5^. Accordingly, why the vestibular system uses two distinct peripheral channels to represent self-motion is currently an open question.

Conventional wisdom has been that the vestibular neurons represent sensory input exclusively through firing rate (reviewed in ref. 3). Specifically, previous studies have characterized how the firing rates of vestibular afferents and their target neurons in the vestibular nuclei encode self-motion information and determine motion detection thresholds^4^,^6^,^7^,^8^,^9^. It is however possible that the longstanding paradox regarding the functional role of two distinct channels to represent self-motion mentioned above stems from the fact that other aspects of spiking activity have not been considered. Theory suggests that temporal codes, in which sensory input is instead represented by the precise timing of action potentials, more efficiently represent sensory stimuli than rate codes^10^. In this context, rate coding has been commonly defined as a neural code in which stimulus attributes are encoded by the number of spikes occurring during a time window whose length is determined by the stimulus timescale. In contrast, temporal coding has been defined as a neural code in which stimulus attributes are encoded by the precise timing of spikes within the same time window^11^,^12^,^13^. This then leads to the question of whether the vestibular system takes advantage of precise spike timing to encode self-motion. Temporal precision in action potential firing has been observed in many other sensory systems^14^,^15^,^16^,^17^,^18^,^19^,^20^ and could, in theory, also exist in the vestibular system^4^. However, no study to date has directly tested whether self-motion information is represented by precise spike timing.

Here we explicitly tested whether neurons in early vestibular pathways use precise spike timing to represent self-motion. The recordings were made from vestibular afferents while monkeys experienced repeated trials of naturalistic self-motion stimuli. We found that, while regular afferents primarily encode motion stimuli through changes in firing rate, irregular afferents instead more reliably discriminated between different stimulus waveforms through differential patterns of precise spike timing. A simple mathematical model reproduced our findings, and provided an explanation of how the nature of the neural code is determined by a balance between neuronal variability and sensitivity. Importantly, afferent target neurons in the central vestibular nuclei also discriminated between self-motion stimuli through precise spike timing, suggesting that spike timing in higher brain areas ensures accurate self-motion perception and behaviour.

## Results

### Precision of spike timing in the vestibular periphery

To study whether the vestibular system uses precise spike timing to represent self-motion information, single-unit recordings were made from peripheral semicircular canal afferents (*N*=22 regular and *N*=35 irregular) and their central target neurons within the vestibular nuclei (*N*=24). We applied naturalistic self-motion stimuli, and first quantified the information encoded through changes in firing rate. We then investigated whether spike timing represents self-motion information. Such an encoding strategy is, by definition nonlinear, and requires reliable and precise spike timing responses. Thus, if early vestibular pathways use spike timing to represent self-motion information, the following three conditions must be met: (1) neurons should respond nonlinearly to naturalistic self-motion stimuli, (2) neurons should display low trial-to-trial variability in their responses to repeated presentations of the same stimulus, and (3) different self-motion stimuli should evoke distinctive and precise patterns of action potentials^11^. Accordingly, we tested whether all three conditions were met by recording vestibular neural spiking responses to repeated trials of naturalistic self-motion and then establishing whether precise spike timing could be reliably used to discriminate between different stimulus waveforms (see the ‘Methods' section).

### Information transmission via changes in firing rate

**Figure 1:**
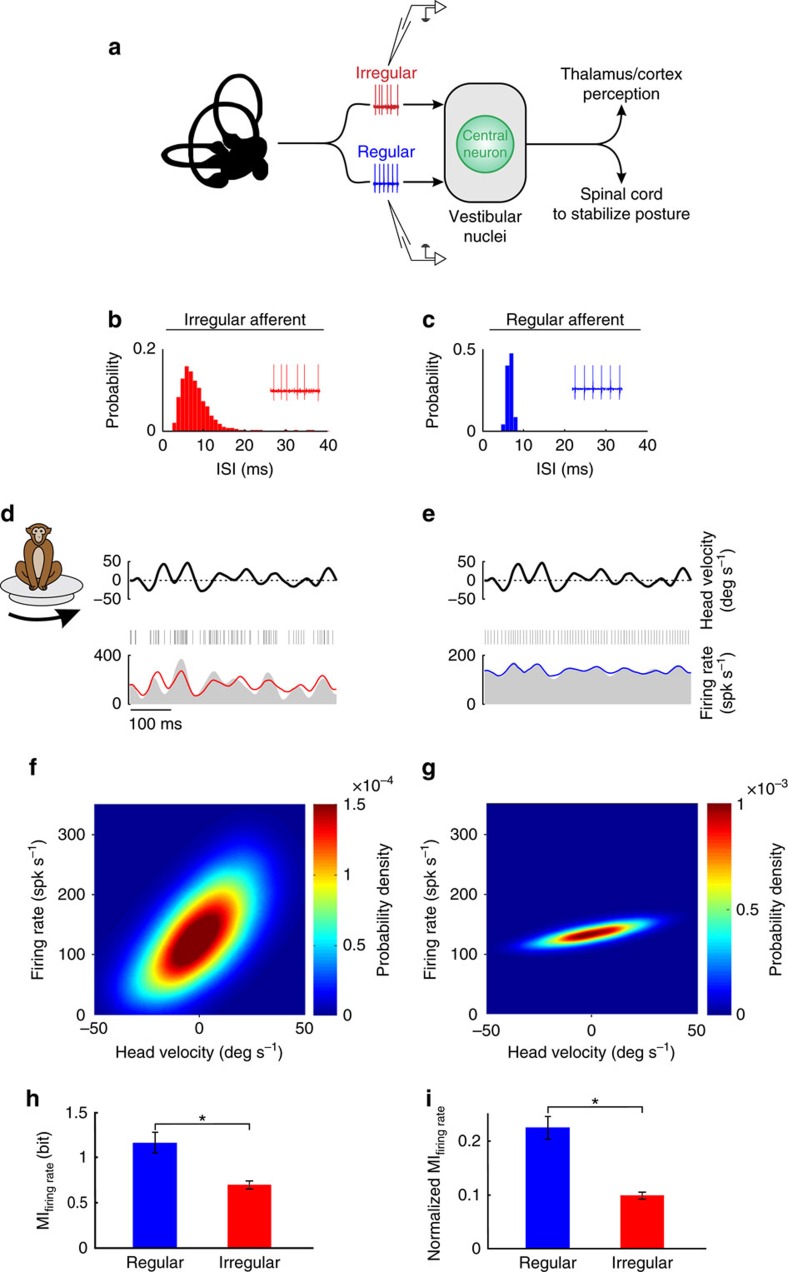
Regular afferents transmit more information through changes in firing rate than irregular afferents. (**a**) Schematic showing early vestibular pathways and recording sites. Single unit recordings were made from individual regular and irregular afferents that project to central neurons within the vestibular nuclei. These central neurons project to the thalamus and cortex and mediate self-motion perception, as well as to the spinal cord for posture stabilization. (**b**,**c**) Interspike interval probability distribution from example irregular and regular afferents, respectively. Insets: spiking activity from these units. (**d**,**e**) Spiking (middle) and firing rate (bottom) response of example irregular and regular afferents to a time-varying head velocity stimulus (top), respectively. The linear firing rate predictions for the irregular and regular afferents are also shown in red and blue, respectively. (**f**,**g**) Firing rate probability density for example irregular and regular afferents, respectively. (**h**) Population-averaged mutual information transmitted by firing rate for regular (blue, *N*=22) and irregular (red, *N*=35) afferents (*P*=0.0028). (**i**) Population-averaged normalized mutual information for regular (blue, *N*=22) and irregular (red, *N*=35) afferents (*P*=1.6 × 10^−5^).

Vestibular afferents display a wide range of resting discharge variability and are typically classified as either irregular or regular (Fig. 1a–c). We applied single presentations of time-varying naturalistic stimuli and found that both irregular and regular afferents responded through changes in firing rate (Fig. 1d,e). Notably, firing rate modulations were markedly greater for irregular afferents due to their greater sensitivity (see Supplementary Fig. 1A for population averages), consistent with the results of previous studies that used artificial stimuli (that is, single sinusoids and band-pass noise) to characterize these cells^4^,^5^. If the increased variability of irregular afferents reduces their information transmission through changes in firing rate (that is, rate coding), we hypothesized that they should encode less information during natural stimulation. Indeed, using the direct method^21^ and computing firing rate conditional probability density as a function of head velocity (see the ‘Methods' section), we found that this was the case. The firing rate probability density showed a marked increase as a function of head velocity and displayed higher variance for irregular afferents as compared with their regular counterparts (compare Fig. 1f,g). Nevertheless, as shown for each population in Fig. 1h,i, regular afferents transmitted significantly higher information than irregular afferents through changes in firing rate (*P*<0.01). Thus, consistent with previous results obtained using an indirect method^4^ (Supplementary Fig. 1B), regular afferents transmitted more information about natural stimuli through changes in firing rate than irregular afferents. We further note that subdivision of irregular afferents into two subgroups corresponding to putative morphological origin^22^,^23^ did not alter the qualitative nature of this finding (Supplementary Fig. 2).

### Irregular afferents display nonlinear responses

**Figure 2:**
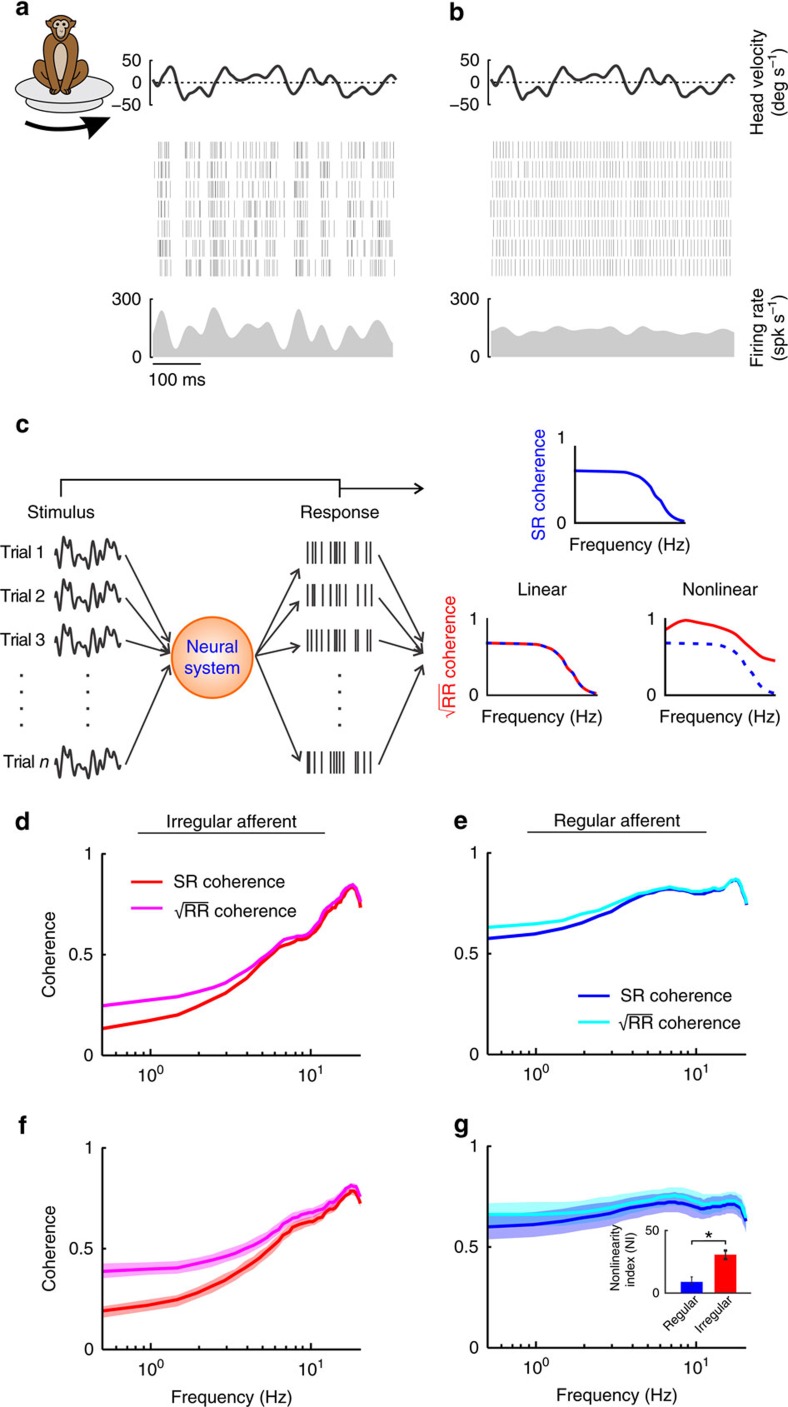
Irregular afferents display nonlinear responses to repeated stimulus presentations. (**a**,**b**) Spiking (middle) and firing rate (bottom) responses of the same example irregular and regular afferents shown in Fig. 1 to repeated stimulus (top) presentations, respectively. (**c**) Schematic showing repeated presentations of the same stimulus and different spiking responses to each trial. The stimulus–response (SR) coherence (blue) measures correlations between stimulus and response (top). In contrast, the response–response (RR) coherence (red) measures correlations between responses to repeated stimulus presentations (bottom). (**d**,**e**) SR (red and blue) and square-rooted RR (purple and cyan) coherence curves obtained for the same example irregular and regular afferents shown in **a** and **b**, respectively. (**f**,**g**) Population-averaged SR (red and blue) and square-rooted RR (purple and cyan) coherence curves obtained for irregular and regular afferents, respectively. Shaded bands illustrate s.e.m. Inset: population-averaged nonlinearity index for regular (blue, *N*=22) and irregular (red, *N*=35) afferents (*P*=6.3 × 10^−6^). ‘*' indicates statistical significance at the *P*=0.05 level using a Wilcoxon rank-sum.

The responses of typical irregular and regular afferents to repeated presentations of naturalistic stimuli are shown in Fig. 2a,b, respectively. Irregular afferents appeared to exhibit more reliable spiking responses across trials than their regular counterparts, leading us to hypothesize that irregular afferents use precise spike timing to represent self-motion. If this is the case, then they should respond nonlinearly to repeated trials of naturalistic self-motion stimuli (that is, condition 1 above). We tested this hypothesis by comparing the stimulus–response and response–response coherence curves for each afferent class (Fig. 2c), as a difference between both is indicative of response nonlinearity^24^. We quantified the difference through a nonlinearity index (NI) that was obtained by integrating the coherence measures over the stimulus frequency range and taking the ratio between the two (see the ‘Methods' section for details). NI is null when the response-response and stimulus-response coherence curves are equal, and approaches 100% with increasing levels of nonlinearity. Indeed, consistent with our prediction, the example irregular afferent showed strong response nonlinearity (Fig. 2d, NI=22%). In contrast, the example regular afferent showed weak response nonlinearity (Fig. 2e, NI=5.7%). Qualitatively similar results were obtained across our data set (Fig. 2f,g, respectively; irregular: 31.5±3.2%, regular: 9.6±2.9%; *P*<0.001), indicating that irregular afferents displayed strongly nonlinear responses to naturalistic self-motion stimuli.

### Irregular afferents use spike timing to encode self-motion

If irregular afferents preferentially use spike timing to represent self-motion information as compared to regular afferents, then there are two additional conditions that must be met: they should display low trial-to-trial variability in their responses to repeated presentations of the same stimulus (condition 2) and different self-motion stimuli should evoke distinctive and precise patterns of action potentials (condition 3). To test whether these conditions were met, we next compared the action potential patterns evoked by different stimuli. Specifically, we quantified the ability of an ideal observer to discriminate between these different stimuli using the recorded spike trains.

**Figure 3:**
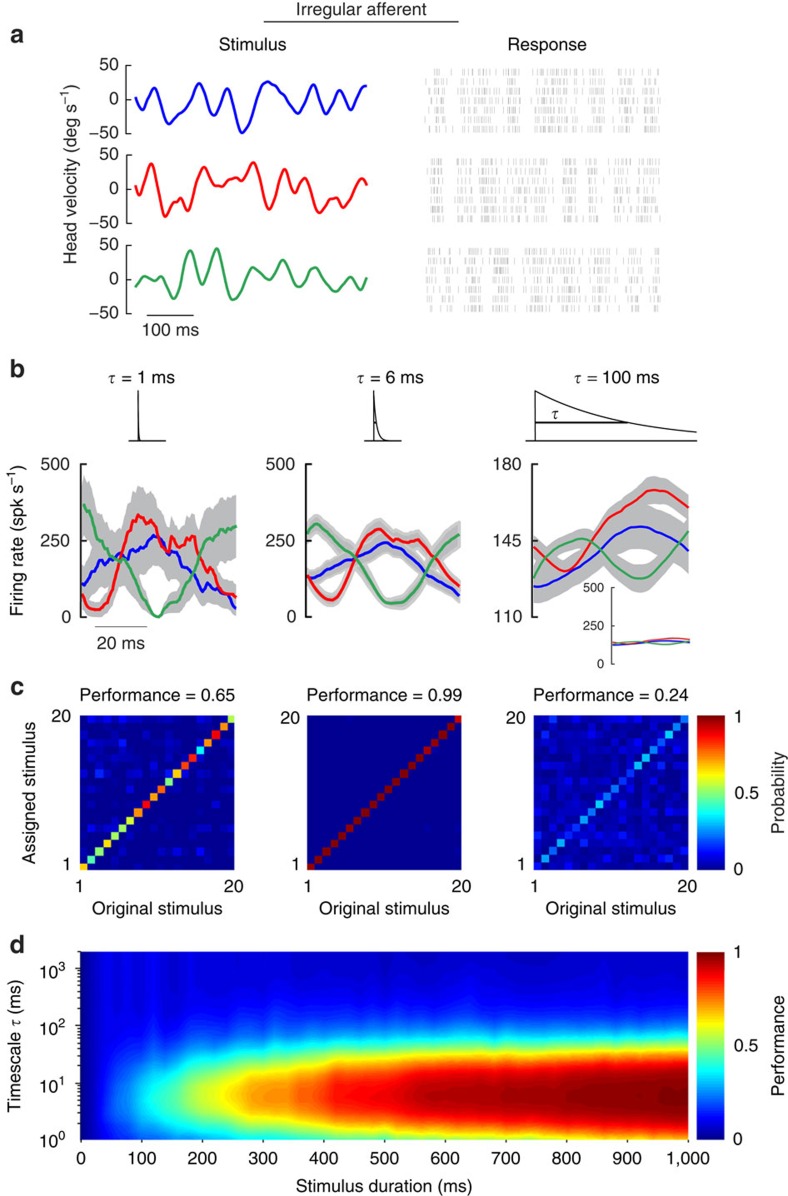
Irregular afferents reliably discriminate between different stimulus waveforms through precise spike timing. (**a**) Spiking responses from an example irregular afferent to repeated presentations of three different stimulus waveforms. (**b**) Average responses with standard error bands (shaded grey) to the three different stimulus waveforms at 1 ms (left), 6 ms (middle) and 100 ms (right) timescales. The responses were obtained by convolving the spike trains with exponential kernels (top insets) that decay with these time constants. To facilitate the comparison the responses are shown using the same scale (right panel, bottom inset). (**c**) Confusion matrices showing the conditional probability of assigning a response caused by stimulus *i* as actually caused by stimulus *j* computed from metric-space analysis using Victor–Purpura measure with timescales (1/*q*) of 1 ms (left), 6 ms (middle) and 100 ms (right). Also shown (top) are the discrimination performance values. (**d**) Performance as a function of the timescale as well as stimulus duration.

Figure 3a shows the spiking responses of the same example irregular afferent described above in Fig. 2 to three different time-varying stimuli with identical statistics (mean=0 deg s^−1^, standard deviation=20 deg s^−1^). Each stimulus was repeated multiple times and we found that, over short timescales (that is, 1 and 6 ms; Fig. 3b, left and middle panels), the variability between responses to different stimuli was often substantially larger than that between responses to the same stimulus (indicated by the grey shading around the three response waveforms). In such cases, stimulus separation was possible and was better at 6ms. In contrast, at longer (for example, 100 ms) timescales, there was significant overlap between responses thereby limiting discriminability (Fig. 3b, right panel). To directly quantify this observation, we used the Victor–Purpura distance metric^25^ and compared the distance between a neuron's responses to a given stimulus and the distance between its responses to different stimuli. Then, to determine whether a given response was correctly predicted as having been elicited by a given stimulus (see the ‘Methods' section), the discrimination performance was computed from the confusion matrix whose element *ij* gives the conditional probability that a response generated by stimulus *i* is classified as being generated by stimulus *j*. Thus, if self-motion is represented by precise spike timing in irregular afferents, then we should expect that they would show maximum discrimination performance at timescales much shorter than those contained in the stimulus.

The confusion matrices obtained from the example irregular afferent are illustrated in Fig. 3c for the same timescales shown in Fig. 3b. Indeed, consistent with our prediction, discrimination performance was maximal for timescales shorter than those contained in the stimulus (Fig. 3c, left and middle panels) and considerably less for longer timescales (Fig. 3c, right panel). Moreover, systematically varying both the timescale and the stimulus duration further revealed that peak discrimination performance was consistently achieved for a timescale of 6 ms (Fig. 3d). Thus, given that this timescale is much shorter than those contained in the stimulus, we conclude that the precise spike timing patterns of this irregular afferent can indeed be used to reliably discriminate between different stimuli.

**Figure 4:**
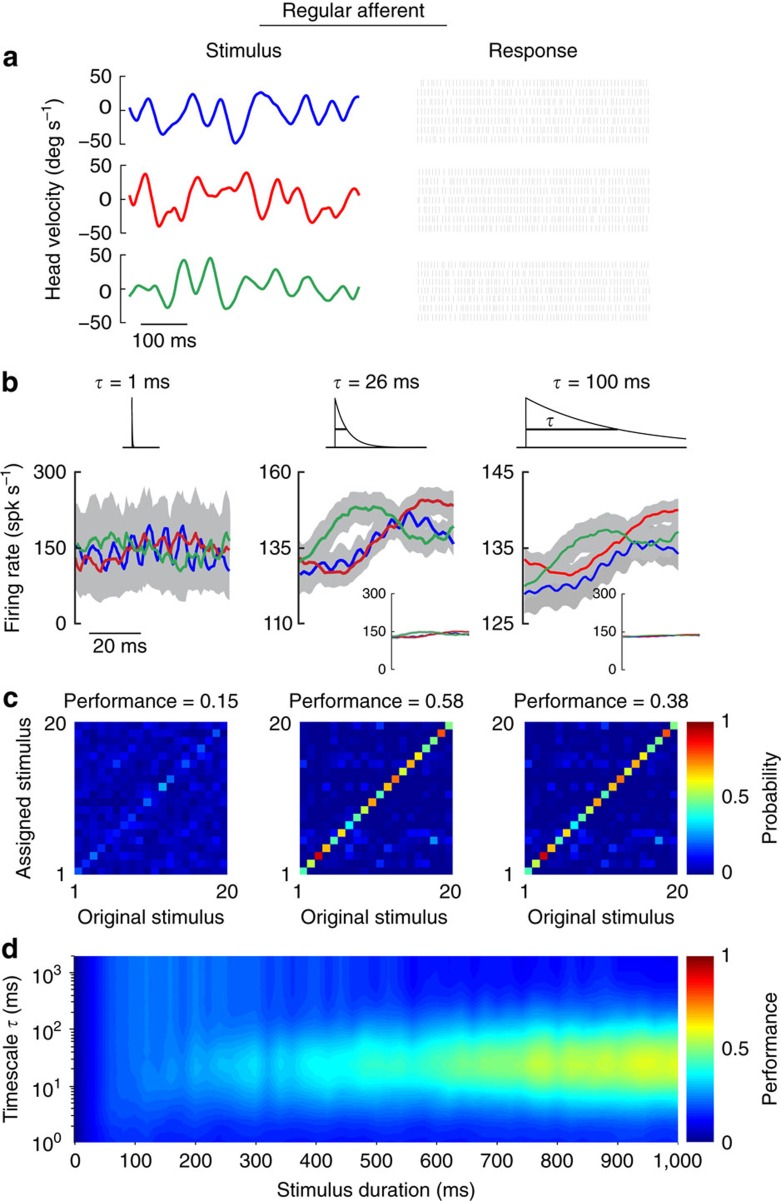
Regular afferents display low performance in discriminating between different stimulus waveforms through precise spike timing. (**a**) Spiking responses from an example regular afferent to repeated presentations of three different stimulus waveforms. (**b**) Average responses with standard error bands (shaded grey) to the three different stimulus waveforms at 1 ms (left), 26 ms (middle) and 100 ms (right) timescales. The responses were obtained by convolving the spike trains with exponential kernels (top insets) that decay with these time constants. To facilitate the comparison, the responses are shown using the same scale (right and middle panels, bottom insets). (**c**) Confusion matrices showing the conditional probability of assigning a response caused by stimulus *i* as actually caused by stimulus *j* computed from metric-space analysis using Victor–Purpura measure with timescales (1/*q*) of 1 ms (left), 26 ms (middle) and 100 ms (right). Also shown (top) are the discrimination performance values. (**d**) Performance as a function of the timescale as well as stimulus duration.

We next performed the same analysis on the example regular afferent (Fig. 4a). In contrast to the irregular afferent shown above in Fig. 3, the regular afferent's responses to different stimuli consistently overlapped for both short (1 ms) and long (100 ms) timescales (Fig. 4b, left and right panels) making stimulus separation difficult. This neuron's responses could be better discriminated at an intermediate timescale (26 ms; Fig. 4b, middle panel) as quantified by performance (Fig. 4c). Indeed, when timescale and stimulus duration were systematically varied, we consistently obtained maximum performance at an intermediate timescale of ∼30 ms (Fig. 4d); a timescale comparable to those contained in stimulus and therefore consistent with rate coding. Furthermore, comparisons between this neuron and example irregular afferent revealed that its performance at short timescales (that is, lower than those contained in the stimulus) was far poorer, and that its maximum performance was actually much lower (compare maximum values in the middle panels of Figs 4c and 3c). In summary, our analysis revealed that unlike the example irregular afferent shown in Fig. 3, our example regular afferent did not represent self-motion through precise spike timing.

**Figure 5:**
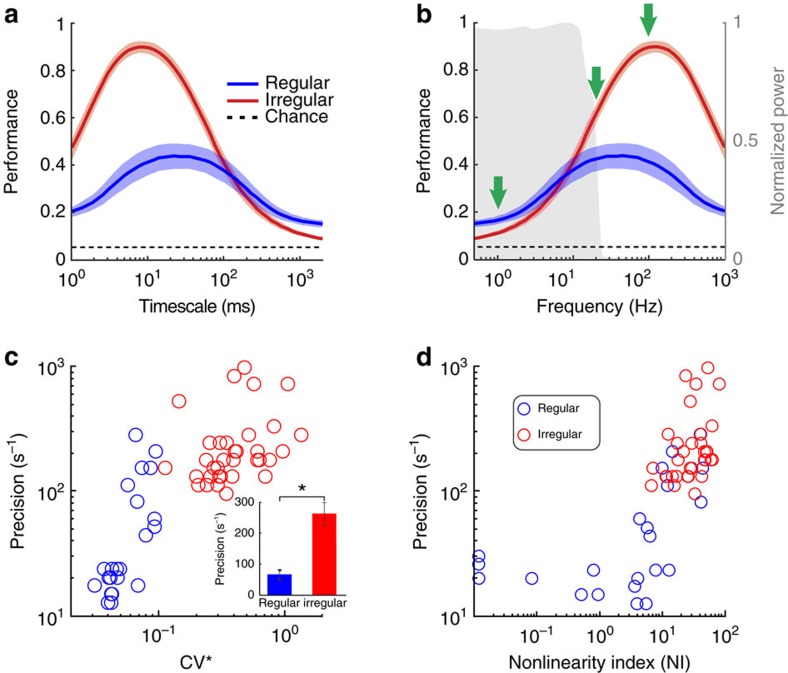
Irregular afferents display higher discrimination performance and greater precision than regular afferents. (**a**) Population-averaged discrimination performance for regular (blue, *N*=22) and irregular (red, *N*=35) afferents as a function of timescale. The shaded red and blue bands show the standard error. (**b**) Population-averaged discrimination performance for regular (blue, *N*=22) and irregular (red, *N*=35) afferents as a function of frequency. The shaded red and blue bands show the standard error. The shaded grey area is the normalized power spectra of the stimulus as a function of frequency. The three arrows highlight the performances at 1, 20 and 100 Hz. (**c**) Spike timing precision as a function of baseline variability as quantified by CV* (see the ‘Methods' section) for regular (blue) and irregular (red) afferents. Inset: population-averaged spike timing precision for regular (blue, *N*=22) and irregular (red, *N*=35) afferents (*P*=4.5 × 10^−7^). ‘*' indicates statistical significance at the *P*=0.05 level using a Wilcoxon rank-sum test. (**d**) Spike timing precision as a function of nonlinearity for regular (blue) and irregular (red) afferents.

Figure 5 illustrates the comparison of the population-averaged results for irregular and regular afferents. Discrimination performance values were consistently greater for irregular than regular afferents at all timescales ranging between 1 and 100 ms (Fig. 5a). Notably, irregular afferents displayed maximum performance at lower timescales (∼6 ms versus ∼30 ms; Fig. 5a, compare red and blue traces) as compared with regular afferents. Accordingly, irregular afferents displayed higher (∼160 Hz) temporal precision (defined as the inverse of the timescale for which performance is maximal) than regular afferents (∼30 Hz). Importantly, the peak performance of irregular afferents was substantially higher than that of their regular counterparts and furthermore occurred at ∼6 ms—a timescale over which the stimulus does not vary significantly.

To better emphasize the implications of this result, Fig. 5b replots the performances of regular (blue trace) and irregular (red trace) afferents as a function of frequency (that is, the inverse of timescale) with the stimulus power spectrum (grey area) superimposed. At lower frequencies (for example, 1 Hz, leftmost green arrow) for which there is significant stimulus power, the performance of regular afferents was greater than that of irregular afferents. This is consistent with our finding above that significantly more information is transmitted by the firing rate of regular versus irregular afferents (Fig. 1h,i). Indeed, the performance of regular afferents approaches its maximum value for frequencies that are contained in the stimulus (that is, for which there is significant stimulus power; ∼20 Hz, middle green arrow). However, this is not the case for irregular afferents. Instead their performance is maximal at a much higher frequency (∼100 Hz) where the stimulus power is negligible. Taken together, the results in Fig. 5b show that, whereas the performance of irregular afferents increases substantially (∼50%) for frequencies greater than those contained in the stimulus (that is, >∼20 Hz and up to ∼100 Hz) or conversely for smaller and smaller timescales (that is, down to ∼10 ms), this was not the case for regular afferents. Instead, there was only a negligible increase in their performance.

Thus, these results suggest that irregular afferents transmit substantially more information through precise spike timing than their regular counterparts. It is important to note that the higher spike timing precision of irregular afferents as compared with their regular counterparts was not due to differences in firing rate. This is because the mean firing rates of regular and irregular afferents in our data set during stimulation were not significantly different from one another (regular: 105±6 spk s^−1^; irregular: 96±6 spk s^−1^, *P*=0.31, tstat=1.03, df=55).

We further found strong positive correlations between spike timing precision (that is, the frequency at which performance is maximal) and baseline variability (Fig. 5c, *R*=0.8, *P*<0.001) as well as response nonlinearity (Fig. 5d, *R*=0.7, *P*<0.001). As expected, a comparable analysis of our data using the van Rossum metric^26^, which has also been commonly used to quantify the distance between spike trains (see the ‘Methods' section, compare Fig. 5a–c with Supplementary Fig. 3), yielded comparable results. Thus, taken together, these results suggest that resting discharge variability strongly influences the nature of the neural code since regular and irregular afferents preferentially use different strategies to encode the sensory stimuli.

### Variability and sensitivity both influence encoding strategy

**Figure 6:**
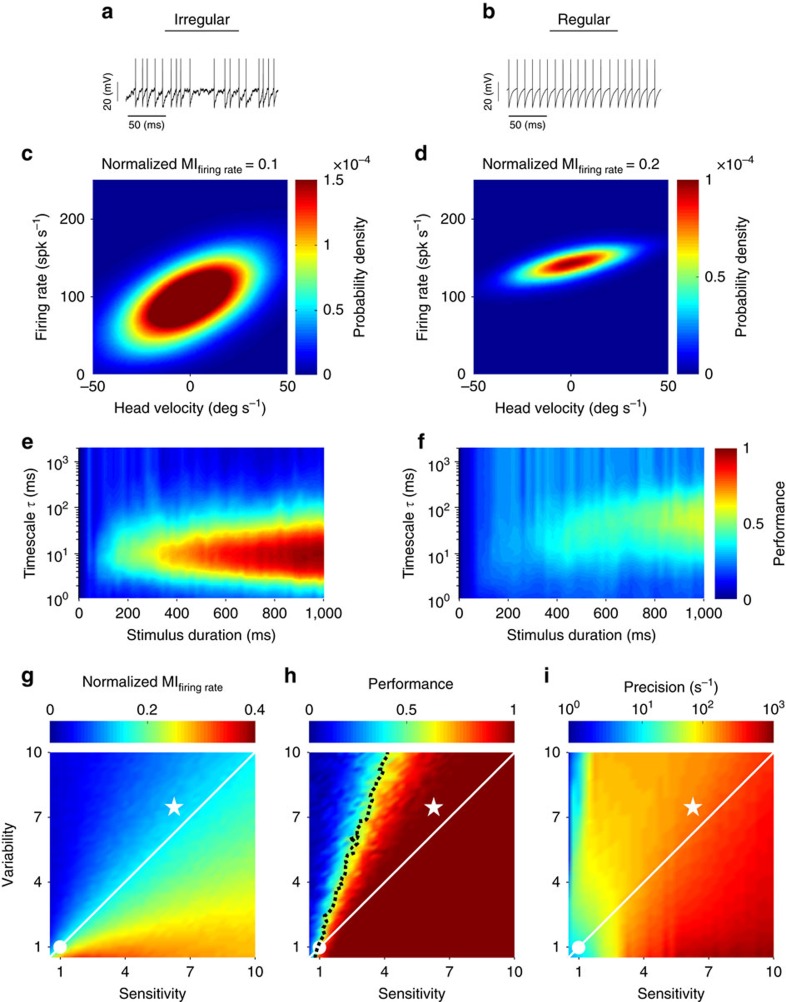
A simple model can explain how variability and sensitivity regulate the nature of neural code in the vestibular periphery. (**a**,**b**) Model irregular and regular afferent spike trains showing high and low baseline variability, respectively. (**c**,**d**) Firing rate probability densities for the irregular and regular model afferents, respectively. The normalized mutual information transmitted by the firing rate of each model afferent is also shown (top). (**e**,**f**) Performance as a function of timescale and stimulus duration for the irregular and regular model afferents, respectively. (**g**) Mutual information transmitted by firing rate as a function of variability and sensitivity for our model. The white circle and star indicate parameter values used for regular (lower left) and irregular (upper right), respectively. Note that the parameter values are normalized such that the variability and sensitivity of the regular model neuron are equal to 1. The solid white line indicates values for which the ratio of variability to sensitivity is equal to that used for the regular model afferent. (**h**) Performance as a function of variability and sensitivity for our model. The dashed black line indicates values for which the performance is equal to that obtained for the regular model afferent. (**i**) Spike timing precision as a function of variability and sensitivity for our model. White circle, star and line: same as in **g** and **h**.

Our results above have shown that afferents with greater intrinsic variability reliably discriminated between different stimulus waveforms through differential patterns of precise (∼6 ms) spike timing, while more regular afferents primarily encoded motion stimuli through changes in firing rate. To gain an understanding of why regular and irregular afferents exhibit different neural coding properties, we built a simple neuron model based on the integrate-and-fire formalism and adjusted parameters so that its spiking output matched experimental data from both afferent classes (Fig. 6a,b, see the ‘Methods' section). In brief, our model comprised a leak term, a bias term that determines the resting discharge, an input current *σ*_signal_ × *S*(*t*), which consists of broadband noise with 20 Hz cutoff similar to the actual head velocity stimuli used in our experiments, and a noise term *σ*_noise_ × *ξ*(*t*) that determines resting discharge variability. We found that this model accurately reproduced our experimental results. Although the model regular afferent transmitted more information through changes in firing rate as compared with the model irregular afferent (Fig. 6c,d), maximum discrimination performance as computed from the confusion matrix was consistently achieved at timescales of ∼6 ms and ∼50 ms for the irregular and regular model afferents, respectively. Thus, consistent with our experimental observations, the model irregular afferent more accurately represented time varying stimuli via precise spike timing than the model regular afferent (Fig. 6e,f).

Why does resting discharge variability influence encoding strategies used by the vestibular periphery? Using this model, it becomes possible to systematically and independently vary sensitivity (*σ*_signal_) and variability (*σ*_noise_). Our results show that higher levels of variability lead to decreased information transmission through firing rate, while increases in neuronal response sensitivity lead to increased information transmission through firing rate (Fig. 6g). Furthermore, our model predicted that the information transmitted will be approximately constant when sensitivity and variability were co-varied such that their ratio (that is, the input signal-to-noise ratio (SNR)) is constant (Fig. 6g, solid white line). Indeed, this finding is consistent with theory because mutual information is determined by the input SNR^10^. As such, our modelling results provide additional strong evidence in agreement with our previous observations, and suggest that the irregular afferents transmit less information through firing rate because they displayed a greater amount of variability relative to sensitivity (compare star with circle in Fig. 6g).

We next quantified the effects of sensitivity and variability on discrimination performance by precise spike timing. Results qualitatively different than those found for information transmission by firing rate were obtained. Specifically, we found that: (i) increasing variability and sensitivity led to decreases and increases in discrimination performance, respectively; (ii) increasing both variability and sensitivity at the same rate actually increased performance (Fig. 6h, solid white line); and (iii) constant discrimination performance was achieved only when variability increased at a rate that was roughly twice that of sensitivity (Fig. 6h, dashed black line). Notably, the timescale for which maximum performance was achieved decreased to values much lower than those contained in the stimulus, which in turn resulted in high precision values (compare Fig. 6h and Fig. 6i). This indicates that co-varying sensitivity and variability while keeping the input SNR constant causes a transition in encoding strategy in which coding by precise spike timing becomes more important (Fig. 6i, white line). Thus, our model shows that both variability and sensitivity can strongly influence the information carried by both firing rate and spike timing, consistent with our experimental results shown above in Fig. 5c,d.

Taken together, our modelling results suggest that irregular afferents represent self-motion through precise spike timing more accurately than their regular counterparts because their increased variability is actually accompanied by a nearly proportional increase in sensitivity. The strong co-variation between variability and sensitivity previously demonstrated experimentally in vestibular afferents^1^,^6^,^27^ is consistent with our modelling results and thus provides support for this proposal.

### Precise spike timing in central vestibular neuron responses

**Figure 7:**
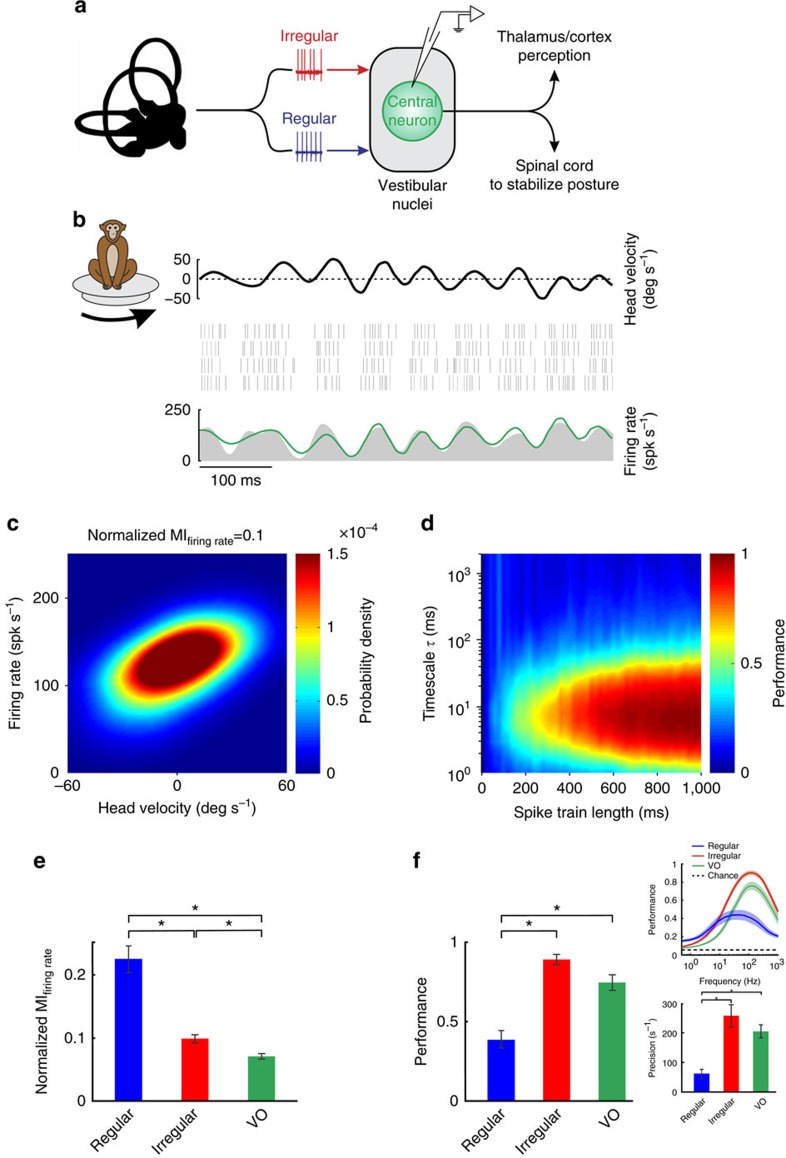
Central vestibular neurons reliably discriminated between different stimulus waveforms through precise spike timing. (**a**) Schematic showing early vestibular pathways. Single unit recordings were made from central neurons within the vestibular nuclei that receive direct input from afferents. (**b**) Spiking (middle) and firing rate (bottom) responses from an example central neuron to repeated stimulus (top) presentations. The linear prediction is shown in green. (**c**) Firing rate probability density for this same neuron with information transmitted by firing rate indicated on top. (**d**) Performance as a function of timescale and stimulus duration for this same neuron. (**e**) Population-averaged mutual information transmitted by firing rate for regular (blue, *N*=22), irregular (red, *N*=35) afferents, and for central vestibular neurons (green, *N*=24; regular–irregular: *P*=1.6 × 10^−5^; regular–central: *P*=2.4 × 10^−6^; irregular–central: *P*=7.5 × 10^−4^). (**f**) Population-averaged discrimination performance by spike timing for regular (blue, *N*=22), irregular (red, *N*=35) afferents and for central vestibular neurons (green, *N*=24). Inset (top): population-averaged performance for regular (blue), irregular (red) afferents and for central vestibular neuron (green) as a function of frequency (top). The shaded bands show the standard error. Inset (bottom): population-averaged spike precision for regular (blue, *N*=22), irregular (red, *N*=35) afferents and for central vestibular neuron (green, *N*=24; regular–irregular: *P*=4.5 × 10^−7^; regular–central: *P*=5.5 × 10^−6^; irregular–central: *P*=0.53). ‘*' indicates statistical significance at the *P*=0.05 level using a Wilcoxon rank-sum test with Bonferroni correction.

Thus far, we have addressed whether the peripheral vestibular system uses precise spike timing to represent self-motion information. We found that afferents with greater intrinsic variability reliably discriminated between different stimulus waveforms through differential patterns of precise spike timing. A priori, if higher brain areas use temporal precision to represent self-motion, then we speculate that postsynaptic central neurons in the vestibular nuclei receiving direct synaptic input from afferents (Fig. 7a) should also demonstrate precise spike timing. Figure 7b illustrates the responses of an example central vestibular neuron to time-varying naturalistic stimuli. The neuron encoded the stimulus through changes in firing rate (Fig. 7b), consistent with previous results^5^,^28^. However, as was the case for irregular afferents, the information transmitted through firing rate was relatively low because this neuron's firing rate conditional probability displayed high variance, (compare Fig. 7c and Fig. 1f with Fig. 1g). It is important to note, however, that further analysis of the precision of this neuron's spiking in response to repeated stimulus presentations revealed strikingly similar results to those obtained above for our analysis of irregular afferents. Specifically, when discrimination performance was quantified from the confusion matrix, we found that maximum performance was consistently achieved at ∼6 ms (Fig. 7d)—a timescale that matches that of irregular afferents (compare with Fig. 3d).

Our analysis of the central neuron population further established that they actually transmitted less information than either regular or irregular afferents through changes in firing rate (*P*<0.001, Wilcoxon rank-sum test with Bonferroni correction; Fig. 7e). However, the discrimination performance from spike timing was comparable to that of irregular afferents (Fig. 7f). Indeed, peak performance was achieved for low timescales (∼6 ms, corresponding to high frequencies of ∼160 Hz for which the stimulus power is negligible; Fig. 7f and Fig. 7f, insets). Thus, we conclude that central vestibular neurons, similar to irregular vestibular afferents, represent self-motion through precise spike timing. This implies that a temporally precise neural representation of self-motion is sent to targets in spinal cord, cerebellum and thalamus to ensure the maintenance of posture and accurate self-motion perception.

## Discussion

Our central finding is that early vestibular pathways use temporally precise firing to represent self-motion. Specifically, we report for the first time that irregular afferents more reliably discriminate between different stimulus waveforms through differential patterns of precise (∼6 ms) spike timing than their regular counterparts. In contrast, regular afferents transmitted more information through firing rate as compared with their irregular counterparts. A simple mathematical model accurately reproduced our experimental data and further explained how variability influences encoding strategies. Furthermore, we found that postsynaptic central vestibular neurons also reliably discriminate between different stimulus waveforms through differential patterns of spike timing, and that the temporal precision of this coding was comparable to that observed for irregular afferents. Taken together, our results indicate that early vestibular pathways use both firing rate and precision of spike timing to represent self-motion. This constitutes a major paradigm shift for the field as previous studies have instead mostly focused on neural responses at relatively (>50 ms) long timescales (reviewed in ref. 3). Moreover, we provide new insight into how the balance between neural variability (a widespread phenomenon across brain structures) and sensitivity can determine encoding strategies.

Conventional wisdom had been that early vestibular pathways use a rate code to encode self-motion information (reviewed in ref. 3). Indeed, this view is supported by numerous studies showing that both afferents and central neurons accurately encode the detailed time course of head rotations through linearly related changes in firing rate over a wide range of frequencies reviewed in refs 1, 5, 29. Moreover, prior investigations had established that both peripheral^30^ and central^28^ vestibular neurons can respond nonlinearly to single repetitions of naturalistic stimuli^1^,^4^. However, while this property is required for temporal coding, it is not sufficient. A major contribution of our study is that it shows that early vestibular pathways use precise spike timing to represent self-motion. Although our results show that self-motion information is present in the precise spike timing of central vestibular neurons, this information must ultimately be decoded to be behaviourally relevant. One possibility is that precise spike timing information is discarded at higher stages of vestibular processing, as observed in the somatosensory system^31^,^32^ (also see refs 17, 18, 20). However, this is unlikely because neurons in higher vestibular areas are actually much less sensitive than the organism^33^,^34^,^35^, suggesting that substantial pooling of neuronal activity must occur to drive perception^5^,^6^,^33^. We further speculate that, at the population level, both irregular afferents and central vestibular neurons will display precisely timed synchronized firing that carries information about naturalistic stimuli, resulting in neuronal thresholds that approach perceptual values. Future studies should focus on how higher vestibular areas (that is, thalamus, cortex) decode precise and potentially synchronized spike timing information.

It is important to note that, under natural conditions, vestibular stimulation results from both active and passive self-motion. Although all the stimuli in the present study were passively applied, previous studies have established that afferents respond similarly to both classes of stimuli^27^,^36^,^37^. Thus, the encoding of self-motion by precise spike timing found in the vestibular periphery is predicted to also be present during active self-motion. However, the central neurons that were the focus of the current study display markedly attenuated responses to active self-motion (reviewed in ref. 3) owing to integration of vestibular and extra-vestibular signals (for example, proprioception and motor). Further studies are needed to uncover whether precise spike timing is also used to represent active self-motion in central vestibular pathways.

Our present results provide the first direct demonstration that precise spike timing of both irregular afferents and central vestibular neurons can be used to reliably discriminate between different stimulus waveforms. We note that a previous study^4^ postulated a seemingly contradictory proposal, namely that regular but not irregular afferents use temporal coding to represent self-motion. This prior study, however, did not record or quantify afferents responses to multiple presentations of the same stimulus. Instead, temporal coding was only indirectly inferred on the basis of the addition of artificial jitter to the spike train response to a single stimulus presentation. In contrast, our present findings directly quantified the precision of spiking to repeated stimulus presentations and directly showed that different spike patterns elicited by different stimuli can be used for discrimination.

By quantifying the precision of the spiking of both irregular afferents and central vestibular neurons to repeated stimulus presentations, we further found that neurons at both stages of processing represent time-varying stimuli through precise spike timing, thereby providing evidence for coding via precise spike timing in early vestibular pathways. In contrast and consistent with previous studies, regular afferents encode self-motion stimuli through changes in firing rate. Taken together, these results indicate that the vestibular nuclei neurons receive two parallel streams of sensory input coded through firing rate and spike timing. Prior studies tracing the projections of the physiologically identified central VO neurons characterized in this report have demonstrated terminations in the spinal cord, consistent with a role in the vestibulospinal reflexes that control posture^38^,^39^,^40^. In addition, VO neurons project to the cerebellum and thalamus^41^,^42^, and are thus thought to play a key role in relaying self-motion information to higher-order areas that contribute to spatial perception and voluntary behaviour. Thus, the major contribution of our study is that it provides the first evidence that the precise spike timing observed in the first two stages of vestibular processing facilitates the discrimination of different self-motion stimuli thus contributing to the control and accuracy of these essential functions.

We posit that parallel streams of afferent sensory input coded preferentially through firing rate and spike timing found at the vestibular periphery are not only preserved but are further refined centrally. Interestingly, previous studies have proposed that central VO neurons primarily receive input from irregular afferents^1^,^3^. Our results showing that both irregular afferents and central VO neurons display similar spike-timing precision is consistent with this proposal. The second primary class of neurons found in the vestibular nuclei, termed position-vestibular-pause (PVP), has a markedly different projection pattern than VO neurons. Specifically, while PVP neurons also receive inputs from vestibular afferents, in contrast to VO neurons, they project to the extraocular motoneurons that control the eye muscles. Accordingly, PVP neurons mediate the vestibulo-ocular reflex (VOR), which stabilizes gaze by moving the eye in the opposite direction to ongoing head motion. Previous studies have shown that the VOR precisely follows and compensates for head motion over a temporal frequency range >25 Hz (ref. 43), implying that detailed information about the stimulus' timecourse is preserved in the VOR pathway. Since such information regarding the detailed patterning of vestibular input is most reliably transmitted through the firing rates of regular afferents^4^, we speculate that PVP neurons preferentially decode and transmit information originating from regular afferents to extraocular motoneurons primarily through changes in firing rate. Further studies investigating the trial-to-trial variability in the spiking responses of PVP neurons across repeated presentations of self-motion stimuli will be required to address this question.

Our simple mathematical model provides an explanation of the mechanism underlying the influence of variability on encoding strategies in early vestibular pathways. Specifically, we found that co-varying sensitivity and variability to keep the input signal-noise relation constant triggered a transition from rate coding to temporal coding (as commonly defined by Theunissen and Miller^11^). By increasing variability and sensitivity, we found a shift from rate coding to temporal coding as quantified by a decrease in discrimination performance through firing rate and an increase in discrimination performance through precise spiking, respectively. Notably, temporal coding on a timescale comparable to that found here for irregular vestibular afferents has been observed across other sensory pathways (visual^15^,^16^,^44^,^45^,^46^, olfactory^47^,^48^, somatosensory^19^,^20^ and auditory^49^,^50^,^51^). However, in contrast to our current findings, these prior studies did not establish the influence of sensitivity and variability on the nature of neural code. This raises the interesting question of whether the mechanism underlying the parallel coding that we observed in early vestibular pathways might similarly mediate the analogous coding by firing rate and spike timing that has been reported in the somatosensory^19^,^20^ and auditory^50^,^52^ systems.

We speculate that the mechanism uncovered in the present study reveals a general feature of neural coding in which a trade-off between sensitivity and variability determine the nature of the neural code. For instance, strong similarities between the vestibular and auditory periphery provide support for common mechanisms. Irregular afferents are more likely than regular afferents to have low voltage-activated potassium currents. Notably these currents, which are critical for determining the characteristics of the membrane recovery time following an action potential^53^,^54^,^55^, are thought to contribute to the increased sensitivity and variability of irregular afferents^2^. Likewise, low voltage-activated potassium currents have been shown to play a key role in regulating precise spike timing in early auditory pathways^56^,^57^,^58^,^59^,^60^. Thus, we hypothesize that similar heterogeneities in intrinsic properties of neurons in the auditory system as well as other sensory pathways are key determinants of the nature of the neural code.

## Methods

All the procedures were approved by the McGill University Animal Care Committee and were in compliance with the guidelines of the Canadian Council on Animal Care.

### Surgical preparation

Two male (*Macaca fascicularis*) and two female (*Macaca mulatta*) macaque monkeys were implanted with a head post for immobilization and recording chambers, which were oriented stereotaxically towards the vestibular nerve and the vestibular nuclei, respectively. The surgical preparation was similar to that previously described^61^.

### Data acquisition and experimental design

We made recordings from two classes of neurons: (1) vestibular afferents that innervate the horizontal semicircular canals, and (2) a group of non-eye movement sensitive neurons in the medial vestibular nuclei, termed vestibular-only (VO) neurons using previously described methodology^28^. Horizontal semicircular canal afferents and VO neurons within the vestibular nuclei were identified as done previously^28^,^37^,^62^. Each neuron was stimulated using a broadband noise angular velocity stimulus (20 Hz cutoff) that had a Gaussian distribution with zero mean and standard deviation of ∼20 deg s^−1^. At least four identical 20 s-long epochs of broadband noise stimulus were concatenated to build a ‘frozen noise' stimulus.

### Analysis of neuronal discharges

Regularity of resting discharge was determined by means of a normalized coefficient of variation (CV*, after Goldberg, Smith^54^) of the interspike intervals (ISIs) recorded during spontaneous activity. Afferents with CV*<0.1 were classified as regular whereas those with CV* ≥0.1 were classified as irregular^27^,^63^. Irregular afferents were further subdivided into two groups based on their response gain at 2 Hz stimulation (high-gain and low-gain) corresponding to putative morphological origin as previously described^22^,^23^ for some analyses.

Neural firing rates fr(*t*) were generated by convolving the spike trains with a Gaussian spike density function (standard deviation of 10 ms) as previously described^64^. Estimates of firing rates were computed using a least-squares regression analysis between the stimulus and filtered spike trains that were aligned with the stimulus waveform as described previously^28^. Note that the mean firing rates during stimulation of regular and irregular afferents were not significantly different (regular: 105±6 spk s^−1^; irregular: 96±6 spk s^−1^; *P*=0.31, tstat=1.03, df=55), consistent with previous characterizations of these neurons (see for example, ref. 27).

The response gain was computed from *G(f)=|P*_*SR*_*(f)/P*_*SS*_*(f)|* where *P*_*SR*_*(f)* is the cross-spectrum between the stimulus *S(t)* and spike train *R(t)*, and *P*_*SS*_*(f)* is the power spectrum of the stimulus *S(t)*. Here *R(t)* is the binary sequence corresponding to the spike train with bin width 1 ms. All spectral quantities (that is, power-spectra, cross-spectra) were estimated using multitaper estimation techniques with eight Slepian functions^65^ as previously described^4^.

We note that the stimuli used in the present study do not elicit simple (that is, rectification, saturation) static nonlinearities in either regular or irregular afferents^5^,^30^. To detect the presence of other nonlinearities in the response, we quantified correlations between the neuronal response *R(t)* and the stimulus *S(t)* using the stimulus-response (SR) coherence *C*_*SR*_*(f)*, as in ref. 24:


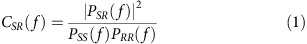


where *P*_*SR*_*(f)* is the cross-spectrum between *S(t)* and *R(t),* and *P*_*SS*_*(f)* and *P*_*RR*_*(f)* are the power spectra of *S(t)* and *R(t)*, respectively. The response–response (RR) coherence between sequences of action potentials was computed by:


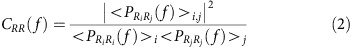


where *P*_*RiRj*_(*f*) is the cross-spectrum between binary sequence *R*_*i*_*(t)* and *R*_*j*_*(t)*, and *P*_*RiRi*_(*f*) and *P*_*RjRj*_(*f*) are the power spectra of *R*_*i*_*(t)* and *R*_*j*_*(t)*, respectively, and <…> denotes the average. For *k* repetitions of the stimuli, the equation above becomes:


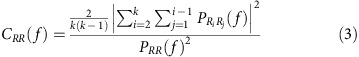


Since in general 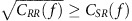, a linear model is optimal if the *SR* coherence equals the square root of the *RR* coherence. A significant difference between these two quantities indicates that a nonlinear model is necessary to explain the relationship between the stimulus *S(t)* and the response *R(t)* for a given frequency *f* (ref. 24). Accordingly, we computed a nonlinearity index (NI) as previously described^66^:


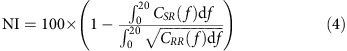


A perfectly linear response results in an NI of zero whereas with increasing non-linearity NI approaches 100%.

To determine the precision of spike timing in the activity of vestibular neurons, and to quantify the timescales at which these neurons operate to encode head velocity, we used metric-space analysis of the spike train. First, we split each 20 s-long epoch of broadband noise stimulus into 20 1 s-long segments (that is, 20 different categories of head velocity stimuli). For each category, one spike train was randomly chosen as a template and the remaining spike trains were assigned to one of 20 categories of stimuli based on the spike distance measure (see below). This procedure was repeated 30 times by drawing different template choices and averages were then computed to construct a confusion matrix (Figs 3c and 4c) whose element (*i*,*j*) gives the probability that a response was assigned as being generated by stimulus *j* given that it was actually generated by stimulus *i*. The diagonal elements of this matrix are the probabilities that a stimulus was correctly assigned, whereas non-zero off-diagonal elements indicate misclassification. For each confusion matrix obtained from the metric-space analysis, we computed the discrimination performance by averaging over the diagonal elements. The discrimination performance can thus vary between 0 (no discrimination) and 1 (perfect discrimination). Note that the chance level for discrimination performance was 0.05 (that is, 1/20) because we used 20 stimuli.

To determine the dissimilarity between two spike trains, we used two well-known measures of spike distance:

The Victor–Purpura metric (VP_spike_) is a cost-based metric that measures dissimilarity between two spike trains based on the minimum cost of transforming a spike train into another spike train through a series of basic operations: insertion and deletion of a single spike are permitted for a cost of 1, and a spike can be shifted by an amount Δ*t* for a cost of *q*Δ*t*^25^,^67^, where *q* (in units of s^−1^) is a parameter that determines the relative sensitivity of the metric to spike count and spike timing^68^. When *q*=0, spike trains are compared under the assumption of a rate code, whereas for high values of *q*, they are compared under the assumption of a temporal code^25^,^67^. The quantity 1/*q* is a measure of temporal precision in this metric; by varying *q* (1≤1/*q*≤2,000 ms) and repeating the classification procedure mentioned above, we investigated the impact of different timescales of the neuronal response on discrimination performance. We assessed the algorithm's performance by constructing the confusion matrix and computing the discrimination performance as described above.

When using the van Rossum spike distance metric, each spike train was convolved with a decaying exponential kernel with time constant *τ*:


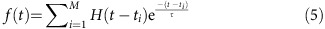


where *t*_*i*_ is *i*th spike time, *M* is the total number of spikes and *H(t)* is the Heaviside step function (*H(x)*=*0* if *x*<*0* and *H(x)*=*1* if *x≥0*). The distance between two spike trains *R*_*j*_*(t)* and *R*_*k*_*(t)* was then defined as the Euclidean distance between their corresponding filtered traces, *f*_*R_j_*_ and *f*_*R_k_*_:


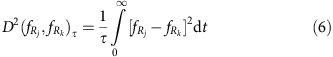


The parameter *τ* is related to the quantity 1/*q* in the Victor–Purpura metric and governs the temporal precision of the metric. Again, we varied *τ* between 1 and 2,000 ms. When *τ* is small, the metric acts as a ‘coincidence detector' since even minor differences in spike timing contribute to the distance, whereas at larger timescales, the difference in total spike count matters, thus the metric becomes more of a ‘rate difference counter'^26^. Note that the qualitative nature of our results does not depend on the specific metric used; (compare Fig. 5 using Victor–Purpura with Supplementary Fig. 3 using van Rossum).

We calculated mutual information between the stimulus and response using the instantaneous firing rate (MI_firing rate_). We computed MI_firing rate_ by first obtaining the instantaneous firing rate fr(*t*) as described above and after adjusting for any time shift between the stimulus and fr(*t*), we plotted the neuron's time dependent firing rate as a function of the shifted stimulus. Next, we used an angular velocity bin-width of 1 deg s^−1^ and a firing rate bin-width of 1 spk s^−1^, to construct a set of stimulus *S* and response *R*. For each stimulus *s*∈*S* and response *r*∈*R*, we determined the conditional probability *p*(*r*|*s*) and then the joint probability *p*(*s*,*r*) by *p*(*s*)*p(*r*|*s*). Finally, the mutual information (MI_firing rate_) between the stimulus set *S* and the response set *R* was computed as^21^:


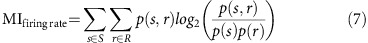


To facilitate the comparison with the discrimination performance obtained using metric-space analysis, we normalized the MI_firing rate_ by the entropy of the response (that is, 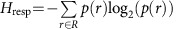) such that:


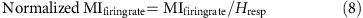


Note that the normalized MI_firing rate_ can vary between 0 and 1.

We built a leaky integrate-and-fire neuron to model the activity of semicircular canal afferents using equations as follows.


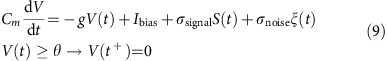


where *C*_*m*_ is the membrane capacitance (*C*_*m*_=1 nF), *V*(*t)* is the membrane potential, *g* is the membrane conductance for the leak current (*g*=0.243 μS), *I*_bias_ is a bias current (to simulate the resting discharge of semicircular canal afferents), *S*(*t*) is the input current, which consisted of a broadband noise current (20 Hz cutoff) similar to the actual head velocity stimuli applied to stimulate the afferents, and *ξ* is a Gaussian white noise process with zero mean and standard deviations of *σ*_noise_. To account for the known response dynamics of both regular and irregular semicircular canal afferents, the stimulus *S*(*t*) used in the model was obtained by filtering the original broadband noise current using the transfer functions of regular and irregular units as done previously^30^. The parameters *σ*_noise_ and *σ*_signal_ determine the response variability and the strength of the signal, respectively. When *V(t)* is greater than or equal to the threshold *θ* (that is, −50 mV), *V(t)* is immediately reset to 0 mV and a spike is said to have occurred at time *t*. Equation (9) was numerically integrated using an Euler–Maruyama algorithm with a time step of 0.025 ms. The spiking responses from the model were analysed in the same way as the experimental data.

For the regular model neuron, parameter values were: *I*_bias_=4.14 nA, *σ*_noise_=0.28 nA, *σ*_signal_=0.58 nA. For the irregular model neuron, parameter values were: *I*_bias_=3.71 nA, *σ*_noise_=2.1 nA, *σ*_signal_=2.9 nA. The parameter values were set such that responses of regular and irregular model neurons mimicked experimental data. We also systematically varied both sensitivity (that is, *σ*_signal_) and variability (that is, *σ*_noise_) in our model and computed information transmitted by firing rate and spike timing as described above for the experimental data. Note that we used the van Rossum metric to minimize computation time as we extensively varied model parameters.

All the values are expressed as mean±s.e.m. Statistical significance was set at *P*<0.05, using Wilcoxon rank-sum tests unless otherwise indicated. To account for multiple comparisons, a Bonferroni correction was applied whenever applicable.

### Data availability

All data supporting the findings of this study are available within the article and the Supplementary Information file.

## Additional information

**How to cite this article**: Jamali, M. *et al*. Self-motion evokes precise spike timing in the primate vestibular system. *Nat. Commun.* **7**, 13229 doi: 10.1038/ncomms13229 (2016).

## Supplementary Material

Supplementary Information Supplementary Figures 1-3
