# Supplementary material for: Self-motion evokes precise spike timing in the primate vestibular system
Source: Nat Commun. 2016 Oct 27;7:13229. doi: 10.1038/ncomms13229 (PMC5095295; doi:10.1038/ncomms13229)
Supplement: Supplementary Information — Supplementary Figures 1-3 [file ncomms13229-s1.pdf]

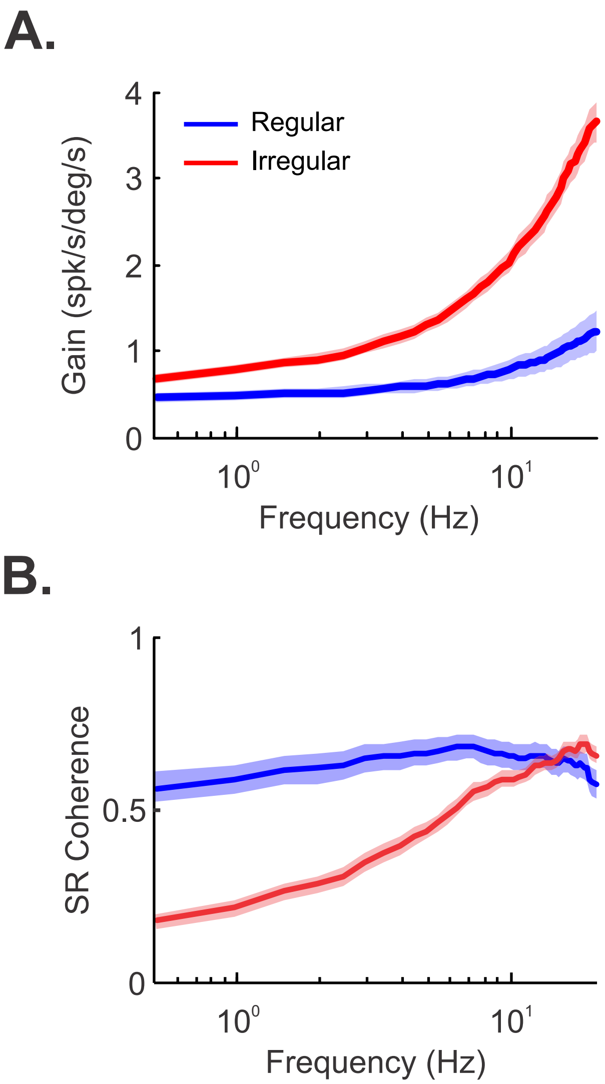

Supplementary Figure 1: *Gain and SR coherence curves for regular and irregular afferents*. **A,B,** Population-averaged gain and stimulus-response coherence obtained for irregular (N=57) and regular (N=38) afferents as a function of frequency. Despite lower gain, regular afferents display higher coherence with respect to the stimuli.

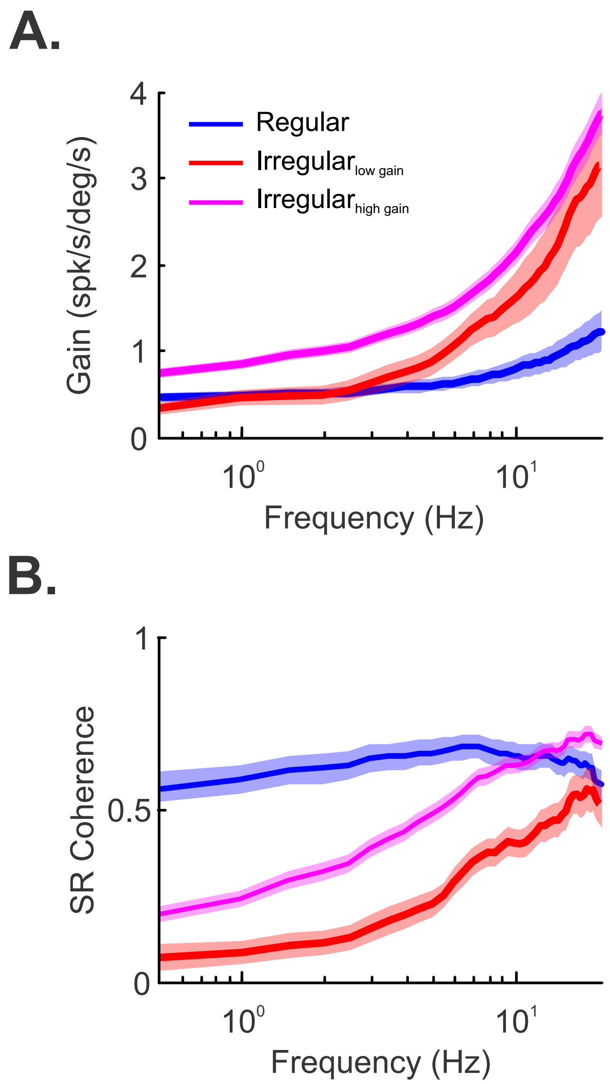

Supplementary Figure 2: *Gain and SR coherence for regular and two groups of irregular afferents*. **A,B,** Population-averaged gain and stimulus-response coherence obtained for low-gain (red, N=10), high-gain (purple, N=47) irregular, as well as regular (blue, N=38) afferents.

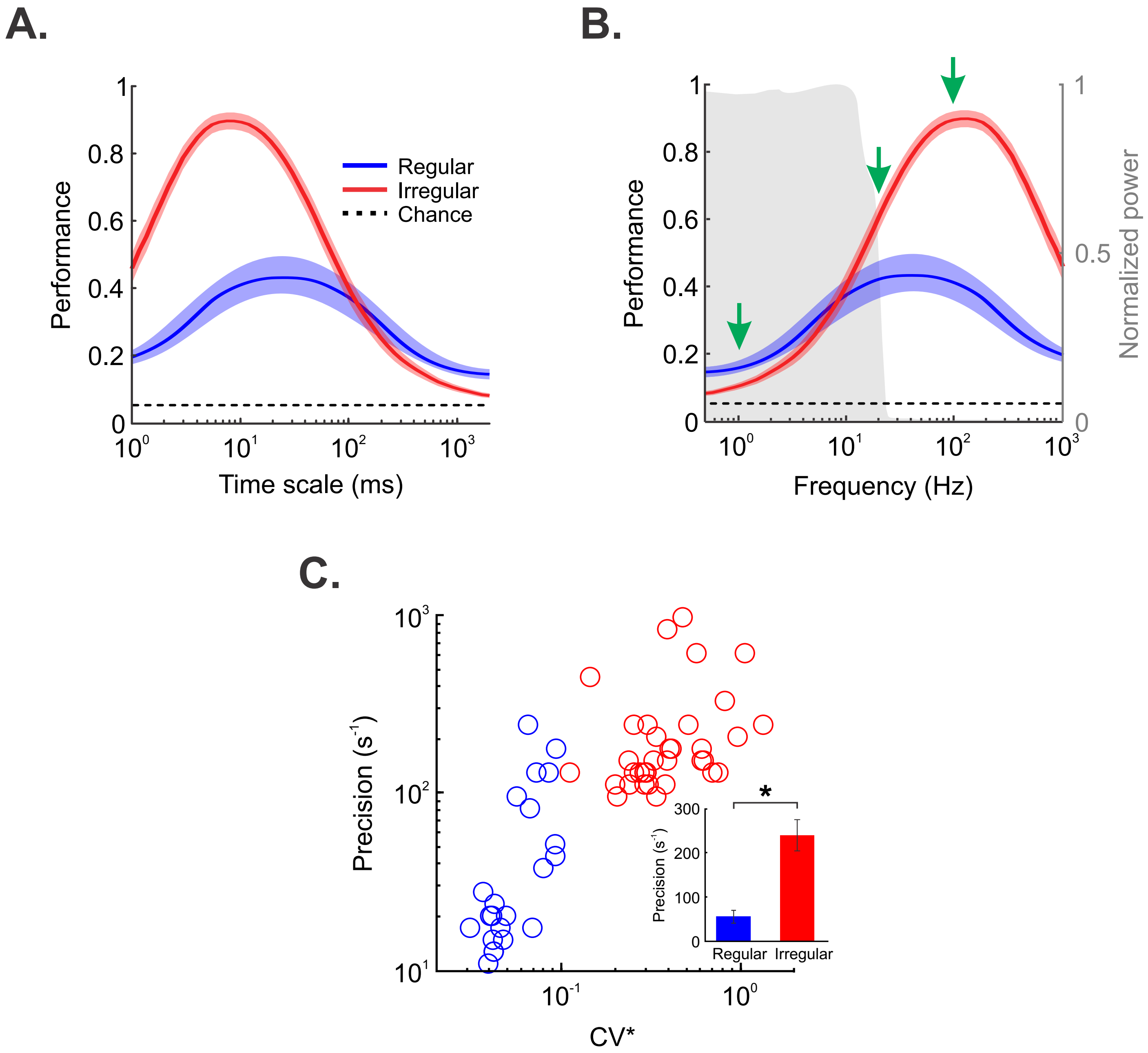

Supplementary Figure 3: *Discrimination performance and precision of spike timing for vestibular afferents using the van Rossum spike train metric.* **A,** Population-averaged discrimination performance through spike timing for regular (blue, N=22) and irregular (red, N=35) afferents as a function of timescale. The shaded red and blue bands show the standard error. **B,** Population-averaged discrimination performance for regular (blue, N=22) and irregular (red, N=35) afferents as a function of frequency. The shaded red and blue bands show the standard error. The shaded grey represents the normalized power spectra of the stimulus as a function of frequency. The three arrows highlight the performances at 1, 20 and 100 Hz. **C,** Spike timing precision as a function of baseline variability as quantified by CV* for regular (blue, N=22) and irregular (red, N=35) afferents. Inset: Population-averaged spike timing precision for regular (blue, N=22) and irregular (red, N=35) afferents ($p=2.6*10^{-7}$). "*" indicates statistical significance at the p=0.05 level using a Wilcoxon rank-sum test.
